# Developing Therapies for Peanut Allergy

**DOI:** 10.1159/000369340

**Published:** 2014-12-20

**Authors:** Merima Bublin, Heimo Breiteneder

**Affiliations:** Department of Pathophysiology and Allergy Research, Medical University of Vienna, Vienna, Austria

**Keywords:** Allergen-specific immunotherapy, Allergen-nonspecific immunotherapy, Ara h 2, Oral immunotherapy, Peanut allergy

## Abstract

Peanut allergy is an IgE-mediated, persisting immune disorder that is of major concern worldwide. Currently, no routine immunotherapy is available to treat this often severe and sometimes fatal food allergy. Traditional subcutaneous allergen immunotherapy with crude peanut extracts has proven not feasible due to the high risk of severe systemic side effects. The allergen-specific approaches under preclinical and clinical investigation comprise subcutaneous, oral, sublingual and epicutaneous immunotherapy with whole-peanut extracts as well as applications of hypoallergenic peanut allergens or T cell epitope peptides. Allergen-nonspecific approaches include monoclonal anti-IgE antibodies, TCM herbal formulations and Toll-like receptor 9-based immunotherapy. The potential of genetically engineered plants with reduced allergen levels is being explored as well as the beneficial influence of lactic acid bacteria and soybean isoflavones on peanut allergen-induced symptoms. Although the underlying mechanisms still need to be elucidated, several of these strategies hold great promise. It can be estimated that individual strategies or a combination thereof will result in a successful immunotherapy regime for peanut-allergic individuals within the next decade.

## Introduction

Peanut allergy is an IgE-mediated disease which tends to develop in early life and resolves in only 20% of peanut-allergic children when they reach school age [1]. Peanut allergy affects 0.8–3% of children and 0.6–0.8% of the adult population in the USA, Canada, UK and Australia [2–4]. So far, the only therapy for peanut allergy is the avoidance of peanuts and peanut-containing foods. Peanut allergens can induce anaphylaxis at minute doses, even in patients who have previously experienced only mild symptoms [5, 6]. Hence, the focus of peanut allergy management is to educate afflicted individuals to avoid all products that contain/may contain peanuts, to recognize early symptoms due to unintended ingestions and to administer self-injectable epinephrine when indicated [7]. However, in one study, these measures were shown to negatively affect the quality of life [8].

Clearly, therapeutic approaches that modify the immune response to peanut allergens and induce oral tolerance are needed as well as strategies that protect the patient from accidental ingestions. Such novel therapeutic approaches for peanut allergy can be generally classified as allergen-specific (table 1) and allergen-nonspecific (table 2) immunotherapies.

## Allergen-Specific Immunotherapy Approaches

Allergen-specific immunotherapy involves subcutaneous injections as well as oral, sublingual or epicutaneous applications of progressively higher doses of the offending allergen over a period of weeks or months to induce clinical desensitization or immune tolerance in individuals with IgE-mediated food allergy. The term ‘clinical desensitization’ is defined as a rise in the threshold dose of an ingested food antigen needed to cause allergic symptoms due to continuous exposure. Oral tolerance refers to the induction of a permanent nonresponse associated with the ability to ingest food without symptoms and without ongoing therapy. While allergen-specific immunotherapy of inhalant allergies has proved to be successful, it is currently not available for clinical use to treat food allergy because of its often limited efficacy which includes the failure to induce oral tolerance and desensitize, the development of serious adverse reactions during therapy and the need for a more prolonged treatment course [9]. Although emerging data from clinical studies support allergen-specific immunotherapy as being effective even in the treatment of severe peanut anaphylaxis, most studies have been conducted with relatively small numbers of subjects and exclude those with severe reactions such as anaphylaxis or severe asthma (table 1). We review here the development of allergen-specific immunotherapy for food allergy in preclinical and clinical studies.

### Subcutaneous Immunotherapy

In contrast to subcutaneous immunotherapy with airborne allergens [10–13], subcutaneous injections of food allergens are associated with unacceptably high rates of severe allergic reactions. Preliminary studies demonstrated the partial efficacy of injection therapy with peanut extract in peanut-allergic patients [14]; this therapy reduced skin-prick test (SPT) reactivity to peanut and increased the tolerance to peanut ingested in double-blind oral challenges [15]. However, systemic reactions occurred both during rush immunotherapy (23%) and maintenance immunotherapy (39%). The study authors concluded that a modified peanut extract was needed for a clinical application of this treatment method [15].

### Oral Immunotherapy

Recent clinical studies of oral immunotherapy (OIT) have made progress toward an active treatment for peanut allergy [16–19]. Early pilot studies demonstrated that peanut OIT was associated with a high incidence of clinical desensitization for the majority of the subjects and was relatively safe when performed by trained personnel in a clinical setting. Furthermore, a gradual updosing regime, including initial-day escalation of the offending food by starting below the eliciting dose followed by a build-up phase consisting of increasing doses in 2-week intervals and then maintenance phases (continued for several months), had a greater positive effect on the safety and efficacy of the treatment than a ‘rush’ protocol which had many side effects and less efficacy during the one-week rush phase [5, 17, 19–22].

Subsequently, peanut OIT was evaluated in 2 randomized, placebo-controlled trials conducted by Varshney et al. [16] at Duke University, N.C., and Anagnostou et al. [18] at Cambridge University Hospitals, Cambridge, UK. In the first of these 2 studies, 28 peanut-allergic children (aged 1–16 years) were randomly assigned to receive OIT for 1 year, gradually escalating the doses of peanut flour up to 4 g/day, or placebo using toastedoats flour [16]. Of 19 children receiving active peanut OIT, 16 (84%) completed the final double-blind, placebo-controlled food challenge (DBPCFC) with 5 g of peanut (equivalent to approx. 20 peanuts) and were considered to be desensitized. In contrast, the 9 placebo subjects could only ingest a median cumulative dose of 0.28 g (equal to approx. 1 peanut) without symptoms. During the initial-day escalation phase, 47% of the peanut OIT subjects experienced clinically relevant side effects requiring antihistamine treatments, and 3 patients with-drew from the study.

In the second study, consisting of 2 phases, 99 peanut-allergic children (aged 7–16 years) were randomly assigned to receive either 26 weeks of OIT using increasing doses of peanut flour up to 800 mg/day or to avoid peanuts [18]. In the second phase, the control patients also underwent the peanut OIT for 26 weeks. A DBPCFC at the end of the study showed that 24 of 39 (62%) children who completed OIT in the first phase and 23 of 45 (54%) who completed OIT in the second phase tolerated 1,400 mg of peanut flour (equivalent to approx. 10 peanuts) and that none of the control group in the first phase reached the target of 1,400 mg. At the lower dose of 800 mg of peanut flour, 84% of the first group and 91% of the second group tolerated the challenge. Most of the participants experienced side effects during the gradual updosing phase, with mild symptoms, oral itching being the most common (81%). The results of these 2 studies suggest that peanut OIT is effective in inducing clinical desensitization in >50% of peanut-allergic children, raising the threshold doses of reaction and conferring protection against at least 10 peanuts, which is more than are likely to be encountered during accidental ingestion.

The first sustained unresponsiveness occurring after peanut OIT was shown in 50% of subjects treated for up to 5 years with 4,000 mg/day [23]. Of 24 peanut-allergic subjects who completed the OIT protocol, 12 passed a challenge with 5,000 mg of peanut protein without symptoms 1 month after stopping OIT. A survey after 3–4 years revealed that none of the subjects reported symptoms with ad libitum peanut consumption. Despite the relatively small number of participants and the lack of a control group, the results of this particular study are promising and demonstrate that the length of the treatment and the antigen dose influence the permanence of the treatment effect.

The mechanisms that contribute to clinical immune tolerance are still under investigation. Immunological changes noted in patients who receive active treatment but not in those receiving placebo include a decrease of basophil activation and mast cell reactivity usually seen in SPT responses, an increase of peanut-specific IgG4 levels [16, 21, 24], a downregulation of Th2 responses with decreased peanut-specific IL-4, IL-5 and IL-2 and increased production of IL-10, IFN-γ and TNF-α [19, 21]. Jones et al. [21] suggested that apoptosis plays a role in OIT by showing the downregulation of many genes involved in apoptotic pathways. Peanut OIT induced changes in specific IgE-binding and IgG4-binding, especially to Ara h 2 epitopes; this was determined by a microarray containing Ara h 1, Ara h 2 and Ara h 3 peptides [24]. While an overall reduction in peanut-specific IgE levels occurred, an expansion of the IgE repertoire was observed. The newly induced IgG4 was directed to the same epitopes that bound IgE prior to peanut OIT [24].

However, a recent study showed that IgE and IgG4 antibodies and basophil activation measurements allowed a distinction to be made between desensitized and control subjects, but did not differentiate between peanut-desensitized and peanut-tolerant subjects [25]. This study, performed with a small cohort, found that clinical immune tolerance in subjects receiving OIT was associated with an increase of antigen-induced T regulatory (Treg) cells specific for the suppression of autologous effector T cells. The data suggested that epigenetic modification of these Treg cells inducing the hypomethylation of cytosine phosphorothioate guanosine (CpG) sites within the forkhead box protein 3 (Foxp3) locus might be predictive of induction of immune tolerance during OIT. The presumed mechanism of action was that dendritic cells (DCs) expressing indoleamine 2,3-dioxygenase might promote the conversion of naïve CD4+ T cells to Treg cells, probably mediated through epigenetic changes in the Foxp3 locus.

Although peanut OIT has demonstrated clinical efficacy, at least for desensitization, it will remain an experimental treatment until aspects like mechanisms of action, dosing regimens and the short- and long-term effects have been established [26–28].

### Sublingual Immunotherapy

An alternative to peanut allergy injection immunotherapy is sublingual immunotherapy (SLIT). The sublingual route of allergen administration is of great interest as it is effective for inducing allergen-specific tolerance in humans with respiratory allergies [29]. The potential advantage of this route is that it allows the food proteins to bypass gastric digestion. In addition, local tolerogenic antigen-presenting cells in the oral mucosa can potentially enhance the induction of tolerance. Applying allergens to the mucosal surface is thought to allow direct contact with and processing of the allergen by local tolerogenic DCs that subsequently migrate to cervical lymph nodes and induce tolerant Th1 and Treg cell responses [30].

Peanut SLIT is performed by administering gradually increasing doses of liquid peanut extracts that are held under the tongue for 2 min prior to swallowing. The protocol, similar to that for OIT, consists of an initial biweekly dose escalation phase and a maintenance phase. The treatment doses typically start with micrograms and increase to 2 mg. This is approximately 1,000 times less than the doses given in peanut OIT (up to 4 g). Dosing options for SLIT are limited to milligrams due to the maximum concentrations of available peanut extracts.

So far, the results of 2 clinical, randomized DBPCFC trials evaluating the efficacy and safety of peanut SLIT have been published. In the first study, 18 children (median age 5.2 years, range 1–11 years) completed a 12-month daily treatment updosed to a maintenance dose of 2 mg of peanut protein. In the oral food challenge that followed, the patients in the active-treatment group consumed a median of 1.7 mg of peanut protein (equal to approx. 7 peanuts) and the placebo group ingested a median of 85 mg before they experienced allergic reactions [31]. In the second study, a multicenter study with a cross-over design, 40 adolescents and adults (median age 15 years, range 12–37 years) were enrolled [32]. The maintenance dose for the active group was 1,386 mg of peanut protein. After 44 weeks of treatment, the placebo-treated subjects crossed over to a daily maintenance dose of 3,696 mg of peanut protein while subjects receiving the active treatment continued with the dose of 1,386 mg for another 24 weeks. After 44 weeks of SLIT, 14 of 20 (70%) patients were able to consume 10 times more peanut protein than at the baseline oral challenge, with no allergic symptoms. The median of the consumed dose without symptoms had increased from 3.5 mg for the baseline challenge to 498 mg after 44 weeks. After 68 weeks, the median increased to 996 mg. From the cross-over group which received a higher maintenance dose, 7 of 16 (44%) subjects were able to consume 10 times more peanut protein than at their baseline oral challenge (an increase from a median of 21 to 496 mg). These results indicated that there was an increased clinical benefit with greater desensitization when a longer treatment course was used. Both studies demonstrated that peanut SLIT was well tolerated. The most common side effects involved only the oral-pharyngeal mucosa. However, the dose reached at the end of the desensitization, approximately 1 g of peanut protein, was much lower than what had been achieved with peanut OIT. A retrospective comparison of SLIT versus OIT for peanut-allergic children found that OIT was more effective for inducing clinical desensitization, whereas SLIT was safer for inducing only mild local events in the oral mucosa [33].

Immunological studies have revealed that active peanut SLIT but not placebo increased peanut-specific IgG4 levels and decreased basophil responsiveness to peanut protein extracts. However, no difference was found between the desensitized individuals and patients who did not respond to the treatment. In contrast, the decrease of the SPT wheal diameter was indicative of the success of desensitization among actively treated patients, suggesting that the desensitization might have been mediated by reduced mast cell reactivity [32]. In addition, SLIT appeared to generate a peanut-specific IgA response. Both salivary and serum levels of peanut-specific IgA increased significantly in subjects receiving SLIT but not in subjects receiving placebo [34]. However, the observed increase in peanut-specific IgA did not correlate with clinical tolerance [35].

Overall, further studies are needed to demonstrate the efficacy of SLIT, particularly with regard to how long after treatment the tolerance lasts. Details about immunological mechanisms still have to be elucidated.

### Epicutaneous Immunotherapy

Another noninvasive method of allergen-specific immunotherapy, i.e. via the epicutaneous route, is currently under active preclinical and clinical investigation. By delivering antigen to the intact skin, epicutaneous immunotherapy (EPIT) is posited to induce a strong immune response while avoiding systemic reactions due to poor vascularization of the epidermis. Allergens presented to intact skin efficiently target epidermal DCs present in the basal and suprabasal layers of the upper epidermis, which, after allergen internalization, migrate rapidly to the draining lymph nodes [36, 37].

Peanut EPIT consists of prolonged and repeated administration of a small amount of peanut allergens delivered through an epicutaneous delivery system that is applied to the skin. An occlusive epidermal delivery system (Viaskin, DBV-Technologies, Paris, France) was used for preclinical and clinical studies on the safety and efficacy of peanut EPIT. Perspiration generated under an occlusive chamber rapidly solubilized the lyophilized allergens from its support and enhanced penetration across the skin by hydration of the stratum corneum, which facilitated diffusion of hydrophilic molecules [36, 38, 39].

Proof-of-principle preclinical studies for the use of EPIT to treat peanut allergy performed in a murine model showed that EPIT reduced the Th2 response, similar to subcutaneous immunotherapy, and allowed desensitization and protection during subsequent oral exposure to peanut [37, 38, 40]. This method was recently tested in humans in an ongoing multicenter, double-blind, placebo-controlled phase-II study in France, which enrolled 54 pediatric patients with a confirmed severe peanut allergy. EPIT was carried out in 28 subjects for 18 months with a daily active application of a patch containing 100 μg of peanut proteins. The rest of the cohort received placebo for 6 months, and then 12 months of active treatment. The success criterium was to reach a cumulative reactive dose 10 times higher than at the beginning of the trial. After 18 months of treatment, 40% of the subjects were desensitized to the targeting cumulative reactive dose. Sixty-seven percent of the responders belonged to a subgroup aged 5–11 years. In this subgroup, a progressive increase in the IgG4 level was also detected [41]. The preliminary results of other ongoing peanut EPIT clinical trials have indicated an acceptable safety profile with mostly mild adverse events at the site of allergen application [9, 42].

These data indicate that EPIT is safe and efficacious for desensitizing peanut-allergic children, at least with regard to increasing the quantity of peanut proteins that can be consumed without symptoms. A recent study also suggested that the induction of Foxp3+ Treg cells by EPIT might establish a long-term tolerance in peanut-sensitized mice [43].

### Recombinant Hypoallergenic Peanut Allergens

The use of recombinant wild-type allergens carries the risk of inducing adverse allergic reactions as a consequence of the allergens’ reactivity with IgE. Therefore, recombinant food allergens need to possess a highly reduced or preferably abolished capacity to bind IgE, in order to ensure a minimal risk of IgE-mediated side effects, while retaining their T cell stimulatory ability. Twelve peanut allergens have been identified so far. They belong to the prolamin (Ara h 2, Ara h 6, Ara h 7 and Ara h 9), cupin (Ara h 1 and Ara h 3), Bet v 1 (Ara h 8), profilin (Ara h 5), glycosyl transferase GT-C (Ara h 10 and Ara h 11) and scorpion toxin-like knottin (Ara h 12 and Ara h 13) superfamilies. The immunodominant linear IgE epitopes of the major allergens Ara h 1 [44], Ara h 2 [45, 46] and Ara h 3 [47] were mapped using overlapping peptides and pooled sera from peanut-allergic patients. Furthermore, the amino acids critical for IgE binding to these linear epitopes were determined by synthesizing wild-type and mutant peptides with single alanine substitutions at each position, followed by the production of hypoallergenic Ara h 1, Ara h 2 and Ara h 3 in *Escherichia coli* [45, 48].

The average decrease in the binding of IgE to the modified allergens compared to the wild-type allergens was 35% for Ara h 1, 71% for Ara h 2 and 41% for Ara h 3. The average stimulation index of peripheral blood mononuclear cells (PBMCs) from peanut-allergic patients induced by the modified allergens in relation to their native counterparts was 72% for Ara h 1, 104% for Ara h 2 and 72% for Ara h 3 [45]. Passive sensitization of RBL-2H3 cells with serum IgE from 2 peanut-allergic patients showed that 1–10 ng/ml of modified (m)Ara h 2 as opposed to 0.1–1 ng/ml of recombinant Ara h 2 were required to elicit a 50% β-hexosaminidase release. Moreover, the mice desensitized with mAra h 2 displayed significantly lower clinical symptom scores and plasma histamine levels than those treated with native Ara h 2 and sham-treated mice [48].

### Immunotherapy with Hypoallergenic Peanut Proteins and Bacterial Adjuvants

Bacterial adjuvants are potent stimulants of Th1 immune responses and can be coadministered to increase the efficacy of peanut vaccines. Heat-killed *Listeria monocytogenes* (HKLM) mixed with the antigen KLH (keyhole limpet hemocyanin) was shown to generate a KLH-specific primary response characterized by the production of Th1 cytokines in mice sensitized with KLH [49]. These results suggested that HKLM might be an effective adjuvant for allergen immunotherapy.

The subcutaneous coadministration of HKLM and a mixture of 3 modified peanut allergens (mAra h 1, mAra h 2 and mAra h 3 [48]) in a murine model of peanut anaphylaxis was shown to reduce plasma histamine levels, peanut-specific IgE synthesis and bronchial constriction after an intragastric peanut challenge when compared with sham-treated mice [50]. Following the intragastric peanut challenge, all mice in the sham- and HKLM-treated groups developed anaphylactic reactions as opposed to only 31% of those in the mAra h 1–3 plus HKLM-treated group. The production of IL-4, IL-5 and IL-13 in the splenocytes was significantly reduced, and IFN-γ production was significantly increased only in mice treated with mAra h 1–3 plus HKLM. Although *L. monocytogenes* is not a particularly invasive organism, it is a pathogen for immunosuppressed patients or in the context of pregnancy.

In another study, the investigators used nonpathogenic *E. coli* cells expressing modified allergens [51]. Similar to the earlier study, following an intragastric peanut challenge, peanut-sensitized mice treated with heat-killed *E. coli* (HKE) expressing mAra h 1–3, i.e. HKE-mAra h 1–3, delivered by the rectal and subcutaneous route had the lowest symptom scores, a reduced production of IL-4, IL-5 and IL-13 by the splenocytes and a long-term down-regulation of peanut allergy when compared to placebo-treated mice. The subcutaneous route was abandoned, because it induced skin inflammation and was unlikely to be acceptable for human use [51]. Following rectal administration of medium (9 μg) and high (90 μg) doses of HKE-mAra h 1–3, the peanut-allergic mice remained protected for up to 10 weeks after treatment.

Finally, the HKE-mAra h 1–3 was renamed EMP-123, a vaccine produced in a GMP facility (Allertein Therapeutics, LLC, Consortium of Food Allergy Research) for use in humans. It consisted of three recombinant modified allergens encapsulated within heat/phenol-inactivated *E. coli* and was tested for safety and immunological effects in a phase-I, nonrandomized, open-label clinical trial conducted in 2 centers [52]. EMP-123 was administered rectally to 10 peanut-allergic adults in weekly dose escalations from 10 to 3,063 μg over 10 weeks, followed by 3 biweekly doses of 3,063 μg. Of the 10 peanut-allergic subjects, 4 experienced no symptoms and 1 had mild rectal symptoms. The other 5 patients experienced adverse reactions which prevented them from completing the trial. The strong potential of the EMP-123 to induce adverse reactions indicated that the IgE-binding epitopes had not been sufficiently modified. The investigation of immunological differences between the 2 patient subgroups showed that median baselines for peanut-specific IgE and Ara h 2-specific IgE, as determined by ImmunoCAP^®^, were significantly higher in the group that did not complete the trial. Basophile activation (at a single dilution of 0.01 μg/ml) and SPT titration with peanut were significantly reduced from baseline in all treated patients, but for IgG4, no difference from baseline values was detected. The authors concluded that, due to the frequency of adverse reactions during the treatment, including anaphylaxis, further modifications of the peanut allergens and/or dosing regimen would be necessary to reduce such reactions [52].

In recent years, Ara h 2 has received the most attention as it is regarded as the most potent peanut allergen and a predictor of clinical reactivity to peanut [4, 53, 54]. Chen et al. [55] and Porterfield et al. [56] found that Ara h 2 and Ara h 6 together accounted for the majority of the effector activity of whole-peanut extract. They demonstrated that Ara h 2 and Ara h 6 were not only major elicitors of anaphylaxis, but that immunotherapy with an Ara h 2/6 fraction alone protected from severe allergic reactions after challenging with peanut [34, 55, 56]. Polysensitization to Ara h 2 and Ara h 1 and/or Ara h 3 appeared to be predictive of more severe reactions while patients with mono-sensitization to Ara h 2 had a significantly lower symptom severity score than polysensitized subjects and a lower level of allergen-specific IgE against peanut extract and Ara h 2 [57–60].

In our recent study, we found that Ara h 1, Ara h 2, Ara h 3 and Ara h 6, members of 2 unrelated protein families, i.e. the 2S albumins and cupins, are highly cross-reactive due to the presence of short, highly similar sequence stretches present on surface-exposed loops [61]. The results of all these recent studies provide considerable new insights into the allergenic potency of peanut allergens and will have an impact on the composition of allergen-specific immunotherapy in the future.

### T Cell Epitope-Based Peptide Vaccines

The use of short synthetic peptides corresponding to sequences of allergen T cell epitopes is an attractive approach to induce immunological tolerance without IgE-mediated adverse reactions. T cell peptide-based vaccines derived from the major allergen Fel d 1 and from phospholipase A_2_ to treat allergy to cat dander or bee venom, respectively, are at various stages of clinical study [74]. However, little has been published in the context of food allergy. Treatment with T cell epitopes of the two major egg allergens, ovomucoid and ovalbumin, administered orally to egg-allergic mice appears to be accompanied by an increased expression of Foxp3 and TGF-β [75, 76]. These results indicate a potential modulation of the T-cell response by induction of allergen-specific Treg cells. The identification of the dominant T cell epitopes of the major peanut allergens is crucial for the development of a similar therapeutic approach for peanut allergy. The dominant T cell epitopes of Ara h 2 [45, 77, 78] and Ara h 1 [79, 80] have been reported and the results hint at the potential use of these peptides to treat peanut allergy in humans.

In the first published studies, 8 distinct Ara h 2 T cell epitopes were identified, but the epitope recognition pattern differed noticeably among patients [77].

Proliferative responses to each of these peptides were also associated with the production of the Th2-type cytokine IL-5, suggesting that these peptides contain epitopes relevant to the pathogenesis of peanut allergy. Subcutaneous or intranasal administration of a vaccine containing the 30 overlapping Ara h 2 20-mers in a C3H/HeJ murine model of peanut anaphylaxis markedly reduced serum Ara h 2-specific IgE and significantly lowered plasma histamine levels and anaphylaxis symptoms scores after challenge [81]. Further, an increased IFN-γ production by splenocytes cultured with Ara h 2 in vitro was observed when compared with controls.

More recently, Prickett et al. [78, 80] identified 5 dominant Ara h 2 and 10 dominant Ara h 1 T cell epitopes which were not recognized by IgE from almost all tested peanut-allergic patients. These T cell epitope peptides were presented by diverse HLA class II types (HLA-DR, HLA-DP and HLA-DQ), emphasizing their broad applicability as candidate peptides for T cell-targeted peptide immunotherapy [78, 80].

### DNA-Based Vaccines

The concept of genetic vaccination was inspired by the observation that the injection of murine muscle cells with naked plasmid DNA resulted in the production of the encoded protein [82]. Studies that followed showed that plasmid DNA immunizations could induce specific humoral and cellular responses to the encoded antigen [83, 84]. As DNA vaccination preferentially induces Th1-skewed immune responses, its application for the prevention and therapy of type 1 allergic diseases has been extensively studied in various mouse models [85].

In 1999, Roy et al. [86] described an oral allergen gene immunization to modulate peanut antigen-induced anaphylactic responses. High-molecular-weight chitosan, a nonimmunogenic mucoadhesive polysaccharide, was complexed with Ara h 2-encoding DNA into nanoparticles which were then fed to mice. The expression of Ara h 2 in the murine intestinal epithelium resulted in the production of allergen-specific secretory IgA and serum IgG2a. When intraperitoneally challenged with Ara h 2, immunized mice showed a substantial reduction of allergen-induced anaphylaxis associated with reduced levels of IgE in comparison to unimmunized mice. In another study, plasmid DNA encoding Ara h 2 was injected intramuscularly into various mouse strains [87]. Ara h 2 or peanut protein injections of immunized C3H/HeJ mice induced anaphylactic reactions whereas immunized AKR/J and BALB/c mice suffered no adverse reactions. The authors suggested that similar interindividual variations would also be likely in humans. The immune response not only depends on the mouse strain but also on the nature of the allergen and the ratio of CpG to the protein expressed [88]. Genetic immunization of SKH-Hr1 mice with Ara h 1 elicited a Th1 response whereas Ara h 3 resulted in a mixed Th1/Th2 response. Increasing the amount of CpG administered, relative to Ara h 3 expressed, reversed the Th1/Th2 response. In contrast, in BALB/c mice, both plasmids produced high IgG1 levels.

Despite the promising results induced by DNA vaccines in various mouse disease models, the immunogenicity of most DNA vaccines tested in humans is low. The data of a phase-I clinical trial with a DNA plasmid vaccine containing the Cry j 2 allergen for the treatment of Japanese cedar allergy suggested that the plasmid vaccine was safe [89].

### Silencing of Allergens in Transgenic Plants

One of the strategies to reduce the incidence of food allergy currently being tested by several research groups is the reduction or abolishment of allergen expression levels in plants by genetic engineering. The method of choice seems to be the RNA interference technology that makes use of RNA-based posttranscriptional gene silencing [90]. As early as 1996, Nakamura and Matsuda [91] described the reduction of the expression levels of several members of the alpha-amylase/trypsin inhibitor family in maturing rice seeds by RNA interference technology. Herman et al. [92] succeeded in completely suppressing the expression of a dominant soybean allergen, Gly m Bd 30K, without any collateral alteration of any other seed protein. Apple plantlets that carried a gene construct for silencing the major apple allergen Mal d 1 produced a significantly reduced SPT response than control plantlets [93]. Accumulation of the profilin Lyc e 1 in transgenic tomatoes could be decreased 10-fold [94] whereas the expression of the allergenic nsLTP Lyc e 3 was decreased to levels below the detection limit, resulting in a reduced SPT reactivity, a trait that was passed on to the next generation of fruits [95].

Allergic reactions to peanuts can be triggered by exposure to very small amounts of several allergenic seed storage proteins. The challenge, therefore, is to produce transgenic plants with complete silencing of the major allergens Ara h 1, 2, 3 and 6. A paper by Dodo et al. [96] reports the first attempt at silencing the production of Ara h 2 in transgenic peanut seeds. The Ara h 2 content was reduced by up to 25% when the crude peanut extracts from randomly selected seeds of the transgenic plants were tested. Due to the heterozygosity of the transgene, frequently only one of the two seeds contained in a pod showed silencing of the Ara h 2 production. The fact that only 50% of the seeds in the first transgenic plant generation inherited the silencing gene construct made these plants unsuitable as a source of hypoallergenic peanuts. The authors expected an increase in the number of pods with two seeds free of Ara h 2 in subsequent generations as the plants become homozygous for the transgene. The feasibility of silencing abundant seed storage proteins such as Ara h 2 and 6 might affect plant health and seed quality [97]. Chu et al. [98] demonstrated that Ara h 2 and Ara h 6 silencing did not affect the expression of other abundant seed proteins including the allergens Ara h 1 and 3. Nor was seedling growth or the resistance of the transgenic plants to *Aspergillus flavus* affected in comparison to the wild-type plants. Moreover, the silencing of Ara h 2 and 6 resulted in a high reduction of IgE-binding.

It has been shown that genetic engineering can enhance the safety of plant foods for atopic individuals without affecting the viability of the plants. Although several proofs of concept now exist, we do not know whether all plant food allergens can be eliminated without negative effects for the plant’s survival.

On the other hand, in order to identify hypoallergenic peanuts, screening of commercially available peanut varieties revealed a natural Ara h 1-deficient peanut variety from Southeast Asia. Mediator release experiments showed almost identical biological activities of Ara h 1-deficient and standard peanut extracts [99]. The authors concluded that the allergenic potential of the Ara h 1-deficient peanut might be compensated by the other peanut allergens.

## Allergen-Nonspecific Immunotherapy Approaches

### Anti-IgE and Cytokine Immunotherapy and Alternative Approaches

Therapeutic approaches such as anti-IgE therapy and cytokine therapy that interfere specifically with the mechanisms of Th2-mediated immunity have been considered for the treatment of peanut allergy. Anti-IgE therapy is based on the concept that an anti-IgE antibody interferes with the binding of IgE to the high affinity FcεRI receptors on mast cells and basophils, thus inhibiting the immune system’s response to allergens by preventing IgE-mediated hypersensitivity reactions. The humanized monoclonal anti-IgE antibody omalizumab is specific for an epitope on the CH3 domain of IgE, a region responsible for binding to both high-affinity (FcεRI) and low-affinity (FcεRII) receptors [100]. Application of omalizumab showed promise in human studies of asthma [101–104], allergic rhinitis [105] and in combination with allergen-specific immunotherapy in patients with grass and birch pollen allergy [106, 107]. Omalizumab (Xolair®, Genentec/Novartis) reduced early- and late-phase allergen-induced responses by decreasing levels of circulating serum IgE [108], the expression of high-affinity IgE receptors [109] and basophile histamine release [110].

A phase-II, multicenter clinical trial treating peanut-allergic patients with omalizumab was discontinued due to concerns for the safety of the patients undergoing oral food challenges [111]. The limited data of the randomized, double-blind treatment showed a potential trend towards an increase of tolerance to peanut in omalizumab- versus placebo-treated subjects. Four of nine (44%) omalizumab-treated patients versus 1 of 5 (20%) placebo-treated patients could tolerate >1,000 mg of peanut flour during an oral food challenge after 24 weeks of treatment. In an earlier study, a similar humanized IgG1 monoclonal anti-IgE antibody (TNX-901) was tested in 84 patients with a history of peanut allergy [112]. In a double-blind, placebo-controlled, randomized trial, patients were subcutaneously injected either with TNX-901 or placebo once every 4 weeks. The efficacy of the treatment was determined within 2 and 4 weeks after the fourth dose on the basis of an oral food challenge. The increase in the mean threshold of sensitivity to peanut at the final oral food challenge (compared with that of the placebo group) only reached statistical significance for the group given 450 mg of TNX-901 (p < 0.001), but a strong trend was associated with increasing doses (p < 0.001). The threshold achieved with a 450-mg dose of TNX-901 was 2,805 mg of peanut flour, which is equivalent to approximately 8 peanuts, an amount that should protect against most unintended peanut ingestions. However, the response to therapy was not uniform, only 21% of patients in the group given 300 mg of TNX-901 and 24% of those who received 450 mg were able to ingest at least 8 g of peanut flour (approx. 24 peanuts), the final dose in the food challenge, before having a reaction. This antibody was not selected for further clinical evaluation.

In a small, uncontrolled pilot study of omalizumab combined with rush peanut OIT, 13 children (median age 10 years) who failed an initial DBPCFC at doses of ≤100 mg of peanut flour were enrolled [113]. After pretreatment with omalizumab, 12 subjects were able to complete the dose escalation phase, continued on a 4-gram maintenance phase, and subsequently tolerated a challenge with 8 g of peanut flour (equivalent to 20 peanuts). However, 7 subjects experienced moderate to severe adverse events during the OIT. These data indicate that the pretreatment of children with high-risk peanut allergy may facilitate oral desensitization. Clinical studies on omalizumab in combination with peanut OIT are ongoing.

A few studies investigated the application of cytokines to inhibit the development of anaphylaxis in peanut-allergic mice. Lee et al. [114] found that liposome-encapsulated recombinant IL-12 administered orally to peanut-allergic mice 3 weeks after sensitization as well as at the time of sensitization attenuated anaphylactic reactions triggered by peanut challenge. This was accompanied by a reduction of peanut-specific IgE and IgG1 and fecal IgA levels. These results also suggest that increases in the ratio of INF-γ to IL-4 and to IL-5 may account for the suppressive effect of IL-12 on peanut-specific IgE and IgG1 production. Further studies will be required to elucidate the mechanisms by which IL-12 suppresses a peanut-specific antibody response. In another study, the investigators examined whether IgE-mediated systemic anaphylaxis was controlled by the in vivo administration of IL-21 using a mouse model of peanut-induced anaphylaxis [115]. The administration of recombinant murine IL-21 or IL-21 expression plasmid prevented anaphylaxis to peanut and suppressed total and peanut-specific-IgE. The authors proposed that IL-21 induced the inhibitor of differentiation 2 protein, which inhibited the class-switch recombination for IgE production.

Liu et al. [116] generated a fusion protein comprising the major peanut allergen Ara h 2 and human inhibitory IgG FcγI. Linking FcγI directly to Ara h 2 causes aggregation of the inhibitory receptor FcγIIb and thus inhibits degranulation of basophils from peanut-allergic patients. The fusion protein also significantly inhibited acute anaphylactic reactions in transgenic mice expressing human FcεRIα and in a murine peanut allergy model.

### Lactic Acid Bacteria and Soybean Isoflavones

Lactic acid bacteria (LAB), some of which are part of the human intestinal microflora, are currently being studied as new mucosal delivery vehicles and adjuvant systems to modulate the allergic immune response [62]. Several clinical studies have shown a positive effect of probiotics on the prevention of atopic diseases [63, 64]. In general, studies from various groups imply that LABs induce cytokine signals that counterbalance allergic Th2 responses (reviewed in [62]). LABs have been coadministered as mucosal adjuvants with the birch pollen allergen Bet v 1 [65] or the house dust mite allergen Der p 1 [66], and they induced a shift towards Th1 immune responses in both cases. Björkstén [67] states that there are currently insufficient data to support the application of probiotics as part of an allergy therapy scheme. The results of different studies vary greatly according to the complexity of the microflora and the genetic background of the host. In addition, the immunomodulatory potential of LABs is highly strain-specific and it is not known to what extent this is relevant between strains of even the same species [68]. A study by de Jonge et al. [69] illustrated this point. In a brown Norway rat model for peanut allergy, only 2 of 8 rats that received *Lactobacillus casei* Shirota along with peanut extract did not show detectable peanut-specific IgE antibodies. This prompted the authors to conclude that *L. casei* Shirota did not downregulate food allergic responses in their animal model of peanut allergy, a finding that should not be taken to imply that other probiotic strains would not have a more desirable effect. In contrast, a soybean-based food supplement, that had been fermented with *Aspergillus oryzae* and LABs and then given to mice after sensitization to peanut, was able to protect against peanut-induced anaphylaxis [70].

Soybeans are the most common source of isoflavones, which possess anti-inflammatory properties [71]. Mice fed with the dietary isoflavones genistein and daidzein prior to sensitization showed significantly reduced anaphylactic symptoms and mast cell degranulation in vivo after peanut challenge compared with mice fed a soy-free diet [72]. The data showing that soybean isoflavones suppress immune sensitization by suppressing DC maturation and subsequent DC-mediated effector cell functions suggest that dietary supplementation with soybean isoflavones could be a novel strategy to prevent the development of allergic reactions to food [73].

### Complementary and Alternative Medicine

TCM (i.e. Traditional Chinese Medicine) herbal formulas have a millennia-long history of use in Asia and are now gaining wider popularity in the West. As food allergy is unknown in the TCM literature, research on TCM herbal therapy for food allergy is still in its infancy and focuses on peanut allergy [117, 118]. Researchers around Xiu-Min Li from the Department of Pediatrics at the Mount Sinai School of Medicine in New York have produced very promising results on the effect and mechanism of a food allergy herbal formula (FAHF) in a mouse model of peanut allergy as well as in a preliminary clinical study. At first, an FAHF-1 containing extracts of 11 herbs was developed [119, 120] and then refined to FAHF-2 which contained extracts of 9 herbs [121]. When FAHF-2 was administered to mice during the experimentally induced development of peanut allergy, peanut-induced anaphylaxis was completely blocked [121]. FAHF-2 was also able to induce tolerance after peanut allergy had been established in a mouse model [122]. Tolerance persisted for at least 4 weeks after treatment and was associated with the modulation of intestinal Th1 and Th2 cytokine production as well as with increased numbers of mesenteric IFN-γ-producing CD8+ cells. Moreover, FAHF-2 protected mice from anaphylactic reactions even after multiple peanut rechallenges for 36 weeks after the discontinuation of the treatment [120]. Reduced peanut-specific IgE and increased IgG2a levels persisted over time. IFN-γ production by CD8+ T cells was markedly increased whereas Th2 cytokine production by CD4+ T cells was reduced by as much as 75%. The long-lasting effect of FAHF-2 represents a therapeutic effect that has not been observed with any other treatment. FAHF-2 is currently being tested by the US Food and Drug Administration as a promising new botanical drug to be used to treat allergies to peanut, tree nut, fish and shellfish [118]. The dose escalation phase I of a double-blind, placebo-controlled clinical trial on food-allergic patients was completed and showed that FAHF-2 was well tolerated [123, 124]. Significantly decreased IL-5 and an increase in culture supernatant IFN-γ and IL-10 levels were found in in vitro studies of PBMCs cultured with FAHF-2 [124]. FAHF-2 reduced allergen-stimulated basophil activation and the percentage of circulating basophils at month 6 of the treatment [123]. FAHF-2 is currently in phase-II safety and efficacy clinical trials.

### Peanut Toll-Like Receptor 9-Based Immunotherapy

Ligands of Toll-like receptor (TLR)9 are immunostimulatory DNA sequence oligonucleotides that contain unmethylated CpG dinucleotides. CpG DNA is a bacterial noncoding DNA, highly enriched in 6 base-pair CpG motifs, that binds with great specificity to its receptor TLR9 which is expressed by the cells of the innate immune system like DCs [125].

It is hypothesized that because TLR9 ligands stimulate innate immunity and have a Th1-biasing effect, they may mimic bacterial exposure and shift the Th2 deviation towards a Th1/Th2 balance. A phase-II clinical study investigating a conjugate of the major ragweed allergen Amb a 1 conjugated to CpG DNA demonstrated the potential for TLR9 vaccines in the treatment of ragweed allergic rhinitis [126].

It was shown that TLR9-deficient mice are resistant to peanut-induced anaphylaxis, indicating a strong imunomodulatory effect of TLR9 on the mucosal immune system [127]. This was associated with a significant decrease of total IgE and peanut-specific IgE and IgA but not of IgG1 or Th2 cytokine production, suggesting a possible deficiency in mucosal isotype switching. DC TLR signaling in response to bacterial extracts or CpG-oligodeoxy-nucleotides (ODNs) alone induced IL-12 production and modified the response to peanut antigens by suppressing T-cell proliferation and Th2 cytokine production [128], indicating that activation of TLR9 may be an approach to immunotherapeutic strategies for peanut allergy.

CpG-ODNs, especially type B, are highly effective in inducing Th1 responses in a therapeutic as well in as a prophylactic approach. In the therapeutic model, coadministration of type-B CpG-ODN plus peanut proteins was highly effective in reducing anaphylactic reactions in mice with established peanut allergy [129]. The therapeutic effect was accompanied by an increase in IFN-γ and peanut-IgG2a, but, in contrast to the previous study, without a significant decrease in peanut IgE [129]. In the same way, a synthetic agonist of TLR9 (Cp-2′-deoxy-7-deazaguanosine or immune modulatory oligonucleotides) [130] was effective in bypassing peanut-induced Th2 immune responses toward Th1 responses accompanied by reduced inflammation in the gastrointestinal tract and anaphylaxis in both the prevention and treatment models.

Similar effects of CpG-ODNs were published previously. Adel-Patient et al. [131] analyzed the effect of the application of peanut protein extract alone or mixed with cholera toxin and CpG-ODN as adjuvants in BALB/c mice orally sensitized to peanut. Oral sensitization to peanut was highly enhanced by a previous subcutaneous application of peanut protein extract, but was prevented by a previous subcutaneous administration of a mix of peanut protein extract, cholera toxin and CpGs.

These results indicate that activation of TLR9 may be an approach to immunotherapeutic strategies for peanut allergy.

## Conclusion

Some of the research discussed here has the potential to result in a feasible therapy for peanut allergy. OIT and SLIT with whole peanut extracts are at present the most promising approaches, but their potential for adverse reactions – although some reports do suggest the achievement of oral tolerance – is still a major concern. While there is evidence that peanut EPIT is safe, further studies are needed to determine its efficacy. Immunotherapy with hypoallergenic mutants combined with bacteria as an adjuvant has been shown to be effective and safe in mice, but is not applicable to humans due to the high rate of adverse reactions. Recent findings illustrating the pre-eminent importance of Ara h 2 for peanut allergy suggest that it is probable that only Ara h 2 and possibly Ara h 6 would be required in a vaccine. However, the IgE-reactivity of each vaccine component will need to be completely abolished before any clinical application can take place. T cell-targeted approaches offer a safe and effective strategy for the specific treatment because the dominant T cell epitopes of Ara h 2 and Ara h 1 as novel candidates for peanut immunotherapy have been determined.

The first genetically engineered peanut plants have shown that the safety of plant-based foods can be enhanced by the silencing of allergen-encoding genes. The investigation of TCM herbal therapy for allergic disorders has become a very active area of research. The formulas are not only well tolerated but FAHF-2 also showed excellent long-term protection.

Anti-IgE therapy trials have been discontinued due to the limited benefit to the participants or to safety concerns. Evidence for the efficacy of probiotics on allergy in humans is scarce. More research in this area is definitely needed to select suitable probiotic strains and to transfer the experience from animal models to humans. Likewise, more research is needed to enable the rational design of allergy DNA vaccines.

Until an efficient therapy becomes available, which might well be a combination of allergen-specific and allergen-nonspecific approaches, the current focus of peanut allergy management still rests with its prevention.

## Figures and Tables

**Table 1:** Allergen-specific immunotherapy approaches for peanut allergy

Approach	Study subjects and inclusion criteria	Immunizing reagent	Effects	Publication
Subcutaneous immunotherapy	Human adults including those with anaphylaxis	Peanut extract	↑ oral tolerance and ↓ SPT; high rate of systemic reactions	1997 [15]
OIT	Children with no history of severe anaphylaxis	Peanut flour	54–84% desensitized to target maintenance doses; side effects in 47 and 81% of treated subjects; ↑ IgG4	2011 [16] and 2014 [18]
SLIT	Children [31] and adults [32] with no history of severe anaphylaxis	Liquid peanut extract	↑ oral tolerance; mild side effects; ↓ SPT; ↑ IgG4 and ↓ basophil activation	2011 [31] and 2013 [32]
EPIT	Children with no history of severe anaphylaxis	Patch containing 100 μg of peanut proteins	↑ oral tolerance in up to 67% mild side effects; ↑ IgG4	2014 [41]
Recombinant hypoallergenic peanut allergens and bacterial adjuvants	Human adults with no history of severe anaphylaxis	mAra h 1–3 plus heat/phenol-inactivated *E. coli*	↓ SPT; ↓ basophil activation; high rate of systemic reactions	2013 [52]
Recombinant hypoallergenic peanut allergens	Murine model of peanut anaphylaxis	mAra h 2	Reduced clinical symptom scores and histamine release	2001 [48]
	RBL-2H3 cells and PBMCs from 4 peanut-allergic patients	mAra h 2	Partially reduced IgE reactivity with retained T cell reactivity	2005 [45]
Peanut extract and bacterial adjuvants	Brown Norway rat model for peanut allergy	Peanut extract plus *L. casei* Shirota	Downregulation of peanut allergic response in 2 of 8 rats	2008 [69]
	Balb/c mice	Peanut extract, cholera toxin and CpG	Prevention of oral sensitization by previous subcutaneous administration of the mix	2007 [131]
	Peanut-allergic C3H/HeJ mice	Modified Ara h 1–3 plus HKLM	Reduced histamine levels, peanut-specific IgE, bronchial constriction and anaphylaxis symptoms	2003 [50]
	Peanut-allergic C3H/HeJ mice	HKE-mAra h 1–3	Reduced production of IL-4, IL-5 and IL-13 by splenocytes and long-term downregulation of peanut hypersensitivity	2003 [51]
T cell epitope-based peptide vaccines	Peanut-allergic C3H/HeJ mice	30 overlapping Ara h 2 20-mers	Reduced histamine release and anaphylaxis symptoms	2007 [81]
DNA-based vaccines	AKR/J mice	Complex of chitosan and Ara h 2-encoding DNA	Reduction of peanut-induced anaphylaxis, reduced level of IgE	1999 [86]
	AKR/J, Balb/c and C3H/HeJ mice	Plasmid DNA encoding Ara h 2	Strain-dependent induction of allergic sensitization	1999 [87]
Hypoallergenic transgenic plants	Western blot with sera from peanut-allergic patients	Seed proteins from transgenic peanut plants with silenced Ara h 2 and Ara h 6	Significant reduction of IgE-binding	2008 [96]

**Table 2:** Allergen-nonspecific immunotherapy approaches for peanut allergy

Approach	Study subjects	Active substance	Effects	Publication (year)
Anti-IgE immunotherapy	Human adults	TNX-901	↑ oral tolerance in up to 24%; no further clinical evaluation	[112] 2003
	Human adults	Omalizumab	↑ oral tolerance; adverse reactions during oral food challenges prior to treatment	[111] 2011
Cytokine immunotherapy	C3H/HeJ mice	Liposome-encapsulated recombinant IL-12	Protection against peanut anaphylaxis; ↓ IgE, IgG1 and fecal IgA	[114] 2001
	AKR/J mice	Recombinant IL-12 or IL-21-expression plasmid	Protection against peanut anaphylaxis; ↓ total and peanut-specific IgE	[115] 2007
TLR9-based immunotherapy	TLR-9-deficient mice	Peanut proteins	Resistance to peanut-induced anaphylaxis; ↓ total and peanut-specific IgE and IgA	[127] 2013
TCM herbal therapy	C3H/HeJ mice	FAHF-1	Protection against peanut anaphylaxis; ↓ mast cell degranulation and histamine release	[119] 2001
	C3H/HeJ mice	FAHF-2	Protection from anaphylaxis lasting up to 36 weeks after treatment; ↓ peanut-specific IgE; ↑ peanut-specific IgG2a	[120] 2009
	Human adults	FAHF-2	Well-tolerated; ↓ allergen-stimulated basophil activation; ↓ percentage of circulating basophils	[123] 2011
Soybean isoflavones	C3H/HeJ mice	Dietary isoflavones genistein and daidzein	↓ anaphylactic symptoms and mast cell degranulation	[72] 2011
